# Persistent atrial fibrillation in heart failure with preserved ejection fraction: Prognostic relevance and association with clinical, imaging and invasive haemodynamic parameters

**DOI:** 10.1111/eci.13184

**Published:** 2019-12-26

**Authors:** Robert Schönbauer, Franz Duca, Andreas A. Kammerlander, Stefan Aschauer, Christina Binder, Caroline Zotter‐Tufaro, Matthias Koschutnik, Lukas Fiedler, Franz Xaver Roithinger, Christian Loewe, Christian Hengstenberg, Diana Bonderman, Julia Mascherbauer

**Affiliations:** ^1^ Department of Internal Medicine II Division of Cardiology Medical University of Vienna Vienna Austria; ^2^ Department of Cardiology Wiener Neustadt Hospital Wiener Neustadt Austria; ^3^ Department of Bioimiging and Image‐Guided Therapy Division of Cardiovascular and Interventional Radiology Medical University of Vienna Vienna Austria

**Keywords:** atrial fibrillation, heart failure with preserved ejection fraction

## Abstract

**Background:**

Atrial fibrillation (AF) is a frequent finding in HFpEF. However, its association with invasive haemodynamics, imaging parameters and outcome in HFpEF is not well established. Furthermore, the relevance of AF subtype with regard to outcome is unclear. This study sought to investigate the prognostic impact of paroxysmal and persistent AF in a well‐defined heart failure with preserved ejection fraction (HFpEF) population.

**Materials and methods:**

Between 2010 and 2016, 254 HFpEF patients were prospectively enrolled. All patients underwent echocardiography as well as left and right heart catheterization. Patients without contraindications underwent CMR including T1 mapping. Follow‐up and outcome data were collected. Patients with significant coronary artery disease were excluded.

**Results:**

A total of 153 patients (60%) suffered from AF, 119 (47%) had persistent and 34 (13%) had paroxysmal AF. By multiple logistic regression analysis, persistent AF was independently associated with NT‐proBNP (*P* = .003), NYHA functional class (*P* = .040), left and right atrial size (*P* = .022 and <.001, respectively), cardiac output (*P* = .002) and COPD (*P* = .034). After a median follow‐up of 23 months (interquartile range 5‐48), 92 patients (36%) reached the primary end point defined as hospitalization for heart failure or cardiovascular death. By multivariate Cox regression analysis, only persistent AF (*P* = .005) and six‐minute walk distance (*P* = .011) were independently associated with the primary end point.

**Conclusions:**

Sixty percent of our HFpEF patients suffered from AF. Persistent but not paroxysmal AF was strongly associated with event‐free survival and was independently related to NYHA functional class, serum NT‐proBNP, atrial size, cardiac ouput and presence of COPD.

## 1 INTRODUCTION

Heart failure is a global epidemic, affecting millions of adults worldwide. The same holds true for atrial fibrillation (AF), which may occur independently from heart failure, but is frequently linked to it.^1^ Almost half of heart failure patients present as heart failure with preserved ejection fraction (HFpEF).^2^ AF is highly prevalent in the HFpEF population, affecting up to 65% of HFpEF patients.^3^ HFpEF and AF share similar risk factors such as arterial hypertension, overweight/obesity, chronic obstructive pulmonary disease (COPD), diabetes and advanced age.^4^ Thus, a close link of HFpEF and AF has already been assumed.^5^, ^6^ However, the confounding factors of AF in HFpEF are largely unknown, and whether AF subtype (persistent versus paroxysmal) is important in terms of prognosis has so far not been investigated.

Increasing left ventricular stiffness is accompanied by LA remodelling, which corresponds to the development of LA fibrosis.^7^ Kosiuk et al have developed and prospectively validated a probability score for the presence of LA arrhythmogenic substrate, based mainly on clinical comorbidities.^8^ Interestingly, factors associated with LA arrhythmogenic substrate were identical to known risk factors for HFpEF such as diabetes, renal dysfunction, advanced age, female gender and hypertension. These findings underline the close association of HFpEF and LA remodelling. LA remodelling and atrial fibrosis are closely related and well‐known risk factor for the perpetuation of AF, that is persistent AF.^8^, ^9^

The present study was designed to investigate the prevalence of paroxysmal and persistent AF in a well‐defined prospective HFpEF cohort and relate it with clinical, imaging and invasive haemodynamic parameters, and with event‐free survival.

## 2 MATERIALS AND METHODS

### 2.1 Study design

This was a prospective observational study performed at the Medical University of Vienna. Between December 2011 and November 2016, consecutive patients with suspected HFpEF were invited to participate. The local Ethics Committee approved the study protocol (EK No. 796/2010). All participants gave written informed consent.

### 2.2 Clinical definitions

HFpEF was diagnosed according to the Guidelines of the European Society of Cardiology^2^ and the American Heart Association.^10^ The following criteria had to be fulfilled: (a) signs or symptoms of heart failure, (b) preserved left ventricular systolic function (ejection fraction ≥50%), (c) N‐terminal prohormone of brain natriuretic peptide (NT‐proBNP) 220 pg/mL and (d) evidence of left ventricular diastolic dysfunction or structural changes (LA enlargement and left ventricular hypertrophy) by transthoracic echocardiography. The haemodynamic diagnosis of HFpEF was confirmed whether the pulmonary artery wedge pressure exceeded 12 mm Hg by right heart catheterization.

Atrial fibrillation lasting <7 days was defined as paroxysmal AF and AF ≥7 days as persistent AF. AF lasting longer than one year was defined as longstanding persistent AF.^4^ Incident AF was diagnosed on follow‐up electrocardiograms.

Reasons for exclusion were invasively confirmed significant coronary artery disease, moderate to severe and severe aortic and mitral valve heart disease irrespective of aetiology as evaluated by transthoracic echocardiography.^11^, ^12^, ^13^ As severe tricuspid regurgitation is a frequent finding in HFpEF,^14^ these patients were not excluded. Other reasons for exclusion were congenital heart disease and cardiac amyloidosis. Screening for amyloidosis was performed according to current recommendations^15^ including transthoracic echocardiography, cardiac magnetic resonance imaging (CMR), ^99m^Tc‐3,3‐diphosphono‐1,2‐propanodicarboxylic acid scintigraphy and, if necessary, endomyocardial biopsy.

### 2.3 Outcome measures

Patients were prospectively followed by ambulatory visits including electrocardiograms and telephone calls at 6‐month intervals. Additionally, electrocardiograms and/or Holter monitoring was performed in case of palpitations. The main outcome measure was a combined end point consisting of hospitalization for heart failure or death from cardiovascular causes. End points were ascertained by follow‐up visits and phone calls and adjudicated by our internal adjudication committee consisting of DB and JM, who were blinded to patient characteristics as well as imaging and haemodynamic data.

### 2.4 Assessment techniques

#### 2.4.1 Transthoracic echocardiography with tissue Doppler analysis

All transthoracic echocardiography studies were performed by board‐certified physicians using scanners such as GE Vivid 7 and Vivid S70 (GE Healthcare). Left ventricular ejection fraction was assessed by biplane Simpson technique. Transmitral inflow was measured by pulsed wave Doppler, septal and lateral e' by pulsed wave tissue Doppler. Signs for severe mitral regurgitation were a vena contracta jet width ≥0.7 cm with a large central regurgitant jet (area >40% of left atrium) or a wall impinging jet of any size accompanied by corresponding quantitative measurements (eg effective regurgitant orifice area ≥40 cm^2^, regurgitant volume ≥60 mL/beat).^11^, ^12^, ^13^

#### 2.4.2 Exercise capacity

For assessment of submaximal exercise capacity, six‐minute walk distance (6MWD) on a 50 metre indoor track was used. For statistical analysis, the percentage of the predicted 6MWD ([6MWD/predicted 6MWD] ×100) was calculated.^16^

#### 2.4.3 Right and left heart catheterization

For right heart catheterization, a 7F Swan‐Ganz catheter (Baxter) was inserted via a jugular or femoral access. Pressures were documented as a digitized mean over the whole respiratory cycle including at least eight consecutive heart cycles using CathCorLX (Siemens AG). In addition to mean pulmonary artery wedge pressure, the systolic, diastolic and mean pulmonary artery pressures were documented. Left ventricular end‐diastolic pressure was manually checked in each patient.

Cardiac output was measured by thermodilution. Furthermore, the transpulmonary gradient was calculated by subtracting wedge pressure from mean pulmonary artery pressure. Diastolic pulmonary vascular pressure gradient was defined as the difference between diastolic pulmonary artery pressure and pulmonary artery wedge pressure during a pullback. Pulmonary vascular resistance was calculated by dividing transpulmonary gradient by cardiac output. Following right heart catheterization, coronary angiography was performed in the same procedure.

#### 2.4.4 Cardiac magnetic resonance imaging

cardiac magnetic resonance imaging examinations were performed on a 1.5 Tesla scanner (MAGNETOM Avanto, Siemens Healthcare GmbH). Patients with an estimated glomerular filtration rate of <30 mL/min/1.73 m^2^ were excluded. CMR was only performed when the heart rate was below 90/min. CMR examinations were performed according to standard protocols^17^, ^18^ including late gadolinium enhancement imaging (0.1 mmoL/kg gadobutrol, Gadovist, Bayer Vital GmbH) and T1 mapping using the modified Look‐Locker inversion (MOLLI) sequence. For pre‐contrast T1 mapping, electrocardiographically triggered MOLLI was applied using a 5(3)3 prototype (5 acquisition heartbeats followed by 3 recovery heartbeats and further 3 acquisition heartbeats). For post‐contrast T1 mapping, a 4 (1) 3 (1) 2 prototype was used. T1 values from a mid‐cavity two‐ and four‐chamber view were averaged. Regions of interest for T1 blood pool values were derived with sufficient distance to papillary muscles and the endomyocardial border.

MOLLI‐extracellular volume (ECV) was calculated according to the following formula^18^:

$$\text{MOLLI - ECV =}\;\left(1-\text{haematocrit}\right)\times \frac{\left(\frac{1}{T1\;\;\text{myo}\;\;\text{post}}\right)-\left(\frac{1}{T1\;\;\text{myo}\;\;\text{pre}}\right)}{\left(\frac{1}{T1\;\;\text{blood}\;\;\text{post}}\right)-\left(\frac{1}{T1\;\;\text{blood}\;\;\text{pre}}\right)}\;$$

T1 myo pre/T1 blood pre indicates myocardial/blood native T1 times, and T1 myo post/T1 blood post indicates T1 times of myocardium/blood 15 minutes after gadobutrol application.

#### 2.4.5 Statistical analysis

IBM SPSS version 21 (SPSS Inc) was used for statistical analyses. For all tests, the significance level was set to *P* < .05. Continuous variables are expressed as mean ± standard deviation or as median and interquartile ranges. Categorical variables are presented as numbers and per cent. Continuous variables were compared using the Mann‐Whitney *U* test, for dichotomous variables the chi‐square test was applied. Parameters were divided into clinical, echocardiographic, CMR and invasive haemodynamic categories. To define factors associated with persistent AF, univariate logistic regression analysis was calculated for each parameter. Significant parameters then entered the multivariate analysis in the respective parameter category. Significance limit to enter this model was *P* < .05.

To assess associations with event‐free survival, separate univariate Cox regression models were performed for all baseline parameters, followed by a multivariate Cox regression model for each parameter category with stepwise forward selection. The significance level for a predictor to enter the model was 0.05, and the limit to stay in the model was 0.1. In a final step, all parameters associated with outcome within the respective group were entered into an additional combined pooled multivariate model using forced entry. Kaplan‐Meier plots (log‐rank test) were applied to verify the time‐dependent discriminative power of paroxysmal and persistent AF on cardiovascular outcome.

## 3 RESULTS

### 3.1 Study population

Between December 2010 and November 2016, 296 HFpEF patients were screened for enrollment. After exclusion of 42 patients [significant coronary artery disease (n = 15), NT‐proBNP <220 pg/mL (n = 14), cardiac amyloidosis (n = 13)], 254 patients were prospectively enrolled. All patients with paroxysmal AF were in sinus rhythm at the time of CMR scan and invasive haemodynamic assessment. On the other hand, all patients with persistent AF were in AF at the time of CMR and invasive haemodynamic assessment.

Baseline characteristics are summarized in Table 1. Mean age was 71 ± 8 years, 70% of study participants were female, and mean body mass index was 30 ± 7. No significant differences were found between the paroxysmal AF and the sinus rhythm cohort. Patients with persistent AF, however, were more frequently male (*P* = .040), presented with worse exercise capacity (*P* = .026), more frequent COPD (*P* = .006), worse functional status (*P* = .003) and higher levels of NT‐pro BNP and gamma‐glutamyl‐transferase (each *P* < .001, respectively). Regarding medical therapy, persistent AF patients were less often on 3‐hydroxy‐3‐methyl‐glutaryl‐coenzyme A reductase inhibitors (*P* = .023) and compared to patients with paroxysmal AF they were also less often on specific antiarrhythmic drugs (*P* < .001). Echocardiography as well as CMR revealed more pronounced LA, right atrial and right ventricular dilatation in persistent AF patients (each *P* < .001, respectively). Furthermore, by CMR these patients had worse right ventricular ejection fractions (*P* < .001) and higher levels of ECV (*P* = .007).

**Table 1:** Baseline characteristics

Variable	Sinus rhythm (n = 101)	Paroxysmal AF (n = 34)	Persistent AF (n = 119)	a *P*‐value
Clinical parameters				
Age, y	70 ± 9	72 ± 10	72 ± 7	.536
Female sex, n (%)	79 (79)	23 (68)	75 (63)	**.040**
Body mass index, kg/m^2^	31 ± 8	31 ± 7	30 ± 6	.299
Diabetes mellitus type II, n (%)	40 (40)	6 (26)	42 (35)	.896
Hyperlipidaemia, n (%)	60 (60)	17 (50)	61 (51)	.379
Arterial hypertension, n (%)	96 (95)	33 (97)	114 (96)	1.000
Heart rate, (beats/min)	71 ± 16	68 ± 10	73 ± 14	.093
% of predicted 6MWD, %	75 ± 26	79 ± 22	67 ± 25	**.026**
Sleep apnoea, n (%)	11 (11)	3 (9)	12 (10)	1.000
COPD, n (%)	28 (28)	6 (18)	48 (40)	**.006**
NYHA functional class, n (%)				
II	45 (45)	14 (41)	29 (24)	**.003**
III	48 (47)	19 (56)	80 (67)	
IV	8 (8)	1 (3)	10 (8)	
NT‐proBNP, pg/mL	560 (360 to 1160)	680 (370 to 1690)	1630 (1050 to 2450)	**<.001**
Glycated haemoglobin, %	6.2 ± 1.1	5.5 ± 1.4	6.2 ± 0.9	.120
eGFR, mL/min/1.73 m^2^	62 (50 to 74)	53 (43 to 72)	58 (43 to 71)	.265
Gamma‐glutamyl‐transferase, U/l	29 (19 to 49)	28 (20 to 50)	52 (33 to 95)	**<.001**
HMG‐CoA reductase inhibitor, n (%)	58 (58)	15 (44)	47 (39)	**.023**
Betablocker, n (%)	74 (73)	26 (76)	91 (76)	.883
Diuretics, n (%)	71 (70)	26 (76)	100 (84)	**.046**
ACE inhibitor, n (%)	27 (27)	9 (26)	41 (34)	.220
AT II rezeptor antagonist, n (%)	42 (42)	11 (32)	41 (34)	.435
Specific antiarrhythmic drugs, n (%)	0 (0)	8 (24)	12 (10)	**<.001** b
Echocardiographic parameters				
LA diameter, mm	59 ± 6	62 ± 9	66 ± 8	**<.001**
LV diameter, mm	43 ± 5	45 ± 6	44 ± 5	.380
RA diameter, mm	58 ± 7	62 ± 9	67 ± 8	**<.001**
RV diameter, mm	35 ± 6	36 ± 7	39 ± 8	**<.001**
Interventricular septum, mm	13 ± 3	13 ± 2	13 ± 3	.116
E/E' ratio	15 (10 to 21)	15 (9 to 20)	13 (10 to 18)	.314
LV ejection fraction, %	60 ± 6	60 ± 6	59 ± 7	.438
Systolic PAP, mm Hg	56 (43 to 71)	51 (44 to 74)	59 (49 to 74)	.117
Cardiac magnetic resonance imaging parameters				
LV end‐diastolic diameter, mm	46 ± 5	51 ± 9	48 ± 5	.136
RV end‐diastolic diameter, mm	38 ± 7	39 ± 7	43 ± 8	**<.001**
Interventricular septum, mm	11 ± 2	11 ± 2	11 ± 2	.179
LA diameter, mm	61 ± 8	63 ± 9	69 ± 9	**<.001**
LA area, cm^2^	27 (23 to 33)	28 (25 to 36)	32 (28 to 37)	**<.001**
RA diameter, mm	61 ± 8	65 ± 8	70 ± 9	**<.001**
RA area, cm^2^	25 (21 to 28)	24 (22 to 28)	33 (27 to 39)	**<.001**
LV ejection fraction, %	67 ± 12	63 ± 10	60 ± 10	**<.001**
LV end‐diastolic volume, mL	118 (102 to 136)	122 (95 to 183)	118 (103 to 142)	.796
Cardiac output, l/min	6.0 ± 3.4	5.7 ± 2.6	5.0 ± 1.5	.078
RV ejection fraction, %	56 ± 12	54 ± 14	48 ± 9	**<.001**
RV end‐diastolic volume, mL	138 (115 to 166)	128 (115 to 179)	148 (116 to 199)	.077
Native T1 time myocardium, ms	426 (371 to 476)	428 (382 to 475)	406 (351 to 460)	.216
MOLLI‐ECV	28.8 ± 3.6	29.0 ± 3.8	30.9 ± 5.0	**.007**
Invasive haemodynamics				
Systolic PAP, mm Hg	52 (39 to 64)	51 (44 to 59)	51 (43 to 64)	.946
Diastolic PAP, mm Hg	21 (17 to 28)	20 (16 to 25)	23 (18 to 27)	.122
Mean PAP, mm Hg	33 (26 to 41)	32 (25 to 38)	34 (29 to 38)	.415
PAWP, mm Hg	19 ± 6	21 ± 6	21 ± 6	.133
LV end‐diastolic pressure, mm Hg	21 ± 7	19 ± 6	20 ± 6	.423
TPG, mm Hg	14 (10 to 19)	12 (9 to 15)	13 (10 to 18)	.779
Diastolic pressure gradient, mm Hg	3.0 (0.0 to 6.0)	0.5 (−2.8 to 3.8)	1.5 (−1.0 to 5.0)	.607
CO thermodilution, l/min	5.5 ± 1.4	5.3 ± 1.3	5.1 ± 1.3	.050
PVR, dyn‐s‐cm^−5^	212 (161 to 278)	198 (119 to 235)	204 (142 to 281)	.884

Note

Variables with a significance level of *p* < .05 are displayed with bold letters.

Values are given as mean ± SD or median and interquartile range or total numbers and per cent.

Abbreviations: 6MWD, 6 min walk distance; ACE, angiotensine converting enzyme; AF, atrial fibrillation; AT II, angiotensin II; CO, cardiac output; COPD, chronic obstructive pulmonary disease; E, early mitral inflow velocity; E', early diastolic mitral annular velocity; eGFR, estimated glomerular filtrationrate; HMG‐CoA, 3‐hydroxy‐3‐methyl‐glutaryl‐coenzyme A; LA, left atrial; LV, left ventricular; MOLLI‐ECV, modified Look‐Locker inversion recovery sequence‐derived extracellular volume; NT‐proBNP, N‐terminalprohormone of brain natriuretic peptide; NYHA, New York Heart Association; PAP, pulmonary arterial pressure; PAWP, pulmonary artery wedge pressure; PVR, pulmonary vascular resistance; RA, right atrial; RV, right ventricular; TPG, transpulmonary pressure gradient.

a *P*‐value indicates the difference between the persistent AF cohort and the rest.

b *P*‐value indicates the difference between the persistent and the paroxysmal AF cohort.

At study enrollment 153 (60%) patients presented with AF. A total of 119 patients (47%) had persistent AF. Of these, 116 (97%) had been diagnosed with AF at least one year prior to enrollment and thus were in longstanding persistent AF. A total of 34 patients (13%) had paroxysmal AF. During the course of the study, four patients developed a new onset of paroxysmal AF. None of these patients experienced a cardiovascular event. Eight patients developed new (direct) onset of persistent AF. Two of them patients experienced a cardiovascular event. However, no close temporal correlation of persistent AF onset and cardiovascular event was observed. In one case, the event of left sided heart failure was 24 months before persistent AF onset, in the other case it was vice versa. Only one conversion from paroxysmal to persistent AF was observed. This patient suffered an episode of left‐sided heart failure 14 months prior the conversion from paroxysmal to persistent AF. Thus, the cumulative incidence of AF over the whole study duration was 165 (65%) with 127 patients (50%) in persistent AF and 38 patients (15%) in paroxysmal AF. Only 16 patients of the AF cohort were on specific antiarrhythmic drugs, namely 13 on amiodarone, 1 on dronedarone and 1 on flecainide. One patient was switched from flecainide to amiodarone during the course of the study.

### 3.2 Factors associated with atrial fibrillation

The association of clinical, imaging and invasive haemodynamic parameters with persistent AF was tested by uni‐ and multivariate logistic regression analyses (Table 2). By multivariate analysis, persistent AF was significantly associated with COPD (*P* = .034), New York Heart Association (NYHA) functional class (*P* = .040), serum NT‐proBNP (*P* = .003), LA (*P* = .022) and right atrial size (*P* < .001) and cardiac output by CMR (*P* = .002).

**Table 2:** Univariable und multivariable logistic regression analysis investigating the association of clinical, imaging and haemodynamic parameters with persistent atrial fibrillation

Variable	Univariate		Multivariate	
	Hazard Ratio (95% CI)	*P*‐value	Hazard Ratio (95% CI)	*P*‐value
Clinical parameters				
Age	1.02 (0.99‐1.05)	.252		
Female sex	0.55 (0.32‐0.95)	**.031**		
Body mass index	0.97 (0.94‐1.01)	.233		
Diabetes mellitus type II	0.96 (0.57‐1.60)	.868		
Hyperlipidaemia	0.79 (0.48‐1.30)	.357		
Arterial hypertension	1.06 (0.32‐3.57)	.924		
% of predicted 6MWD	0.98 (0.97‐0.99)	**.013**		
Sleep apnoea, n	0.97 (0.43‐2.19)	.940		
COPD	2.20 (1.27‐3.82)	**.005**	2.06 (1.06‐4.01)	.034
NYHA class ≥ III	2.29 (1.33‐3.95)	**.003**	2.08 (1.04‐4.18)	.040
NT‐proBNP	1.23 (1.06‐1.42)	**.006**	1.51 (1.15‐1.99)	.003
Glycated haemoglobin	1.15 (0.90‐1.48)	.258		
eGFR	1.00 (0.98‐1.01)	.436		
Gamma‐glutamyl‐transferase	1.01 (1.00‐1.01)	**.006**		
Echocardiographic parameters				
LA diameter	1.12 (1.07‐1.17)	**<.001**		
LV diameter	1.02 (0.98‐1.07)	.354		
RA diameter	1.13 (1.09‐1.18)	**<.001**	1.13 (1.09‐1.18)	<.001
RV diameter	1.08 (1.04‐1.13)	**<.001**		
Interventricular septum	0.93 (0.84‐1.03)	.175		
E/E' ratio	0.95 (0.88‐1.02)	.172		
LV ejection fraction	0.98 (0.93‐1.03)	.420		
Systolic PAP	1.01 (0.99‐1.03)	.249		
Cardiac magnetic resonance imaging parameter				
LV end‐diastolic diameter	1.02 (0.97‐1.08)	.498		
RV end‐diastolic diameter	1.09 (1.04‐1.15)	**.001**		
Interventricular septum	0.92 (0.78‐1.08)	.257		
LA diameter	1.11 (1.06‐1.16)	**<.001**		
LA area	1.09 (1.04‐1.15)	**<.001**	1.12 (1.02‐1.23)	.022
RA diameter	1.13 (1.08‐1.19)	**<.001**		
RA area	1.17 (1.11‐1.24)	**<.001**	1.21 (1.10‐1.33)	<.001
LV ejection fraction	0.94 (0.91‐0.97)	**<.001**		
LV end‐diastolic volume	1.00 (0.99‐1.00)	.431		
Cardiac output	0.79 (0.65‐0.97)	**.021**	0.54 (0.36‐0.79)	.002
RV ejection fraction	0.94 (0.91‐0.97)	**<.001**		
RV end‐diastolic volume	1.01 (0.99‐1.01)	.055		
Native T1 time myocardium	1.00 (0.99‐1.00)	.409		
MOLLI‐ECV	1.12 (1.02‐1.23)	**.017**		
Invasive haemodynamics				
Systolic PAP	1.00 (0.99‐1.02)	.976		
Diastolic PAP	1.03 (0.99‐1.06)	.193		
Mean PAP	1.01 (0.98‐1.04)	.483		
PAWP	1.04 (0.99‐1.09)	.101		
LV end‐diastolic pressure	0.98 (0.93‐1.03)	.347		
TPG	0.99 (0.96‐1.03)	.667		
Diastolic pressure gradient	0.99 (0.95‐1.04)	.793		
CO thermodilution	0.82 (0.67‐1.01)	.066		
PVR	1.00 (1.00‐1.00)	.764		

Variables with a significance level of *p* < .05 are displayed with bold letters.

Abbreviations: 6MWD, 6 min walk distance; ACE, angiotensine converting enzyme; AF, atrial fibrillation; AT II, angiotensin II; CO, cardiac output; COPD, chronic obstructive pulmonary disease; E, early mitral inflow velocity; E', early diastolic mitral annular velocity; eGFR, estimated glomerular filtration rate; HMG‐CoA, 3‐hydroxy‐3‐methyl‐glutaryl‐coenzyme A; LA, left atrial; LV, left ventricular; MOLLI‐ECV, modified Look‐Locker inversion recovery sequence‐derived extracellular volume; NT‐proBNP, N‐terminal prohormone of brain natriuretic peptide; NYHA, New York Heart Association; PAP, pulmonary arterial pressure; PAWP, pulmonary artery wedge pressure; PVR, pulmonary vascular resistance; RA, right atrial; RV, right ventricular; TPG, transpulmonary pressure gradient.

### 3.3 Association of atrial fibrillation with cardiovascular outcome

After a median follow‐up of 23 months (interquartile range 5‐48), 92 patients (36%) reached the combined end point. In nine patients, cardiovascular death was the first event and 83 were hospitalized for acute heart failure.

**Figure 1:**
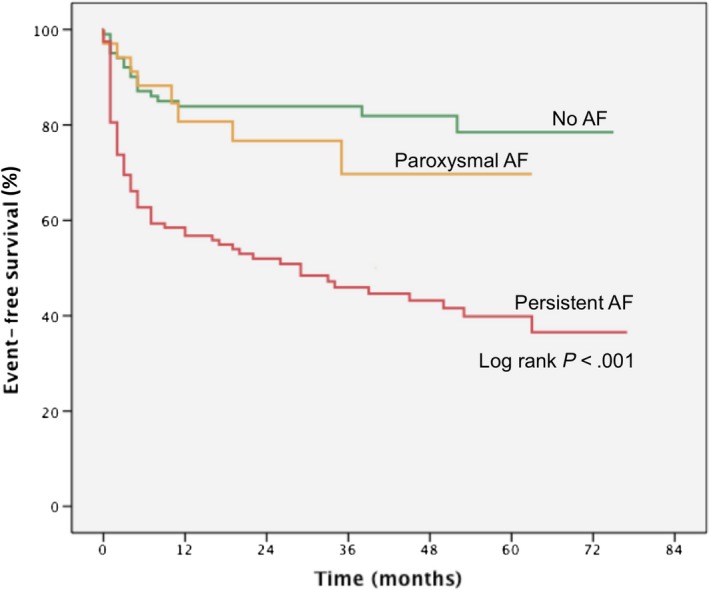
Kaplan‐Meier plot according to atrial fibrillation subtype. Patients with persistent atrial fibrillation had significantly worse event‐free survival rates than those with paroxysmal atrial fibrillation or sinus rhythm. AF indicates atrial fibrillation

Kaplan‐Meier plots showed significantly reduced event‐free survival in patients suffering from persistent AF (log‐rank test *P* < .001). This was not the case for patients with paroxysmal AF (Figure 1).

Table 3 shows the results of the uni‐ and multivariate Cox regression analysis, clustered for clinical, imaging and haemodynamic parameters. Among clinical parameters, persistent AF (*P* < .001), 6MWD (*P* < .001) and reduced estimated glomerular filtration rate (*P* = .030) were significantly associated with outcome in the multivariate analysis. Among invasive haemodynamic parameters, only diastolic pulmonary arterial pressure (*P* = .001) and, among imaging variables, right ventricular size (*P* < .001) and function (*P* = .017) were independently related to event‐free survival.

**Table 3:** Univariable and multivariable Cox regression analyses

Variable	no event (n = 162)	event (n = 92)	Univariate		Multivariate	
			Hazard Ratio (95% CI)	*P*‐value	Hazard Ratio (95% CI)	*P*‐value
Clinical parameters						
Persistent AF, n (%)	53 (33)	66 (72)	3.43 (2.18‐5.41)	**<.001**	3.04 (1.77‐5.24)	<.001
Paroxysmal AF, n (%)	26 (16)	8 (9)	1.39 (0.59‐3.24)	.452		
Age, y	71 ± 9	72 ± 8	1.01 (0.99‐1.04)	.389		
Female sex, n (%)	119 (73)	58 (63)	1.48 (0.97‐2.27)	.070		
Body mass index, kg/m^2^	30.2 ± 6.5	31.0 ± 7.5	1.02 (0.99‐1.05)	.233		
Diabetes mellitus type II, n (%)	46 (28)	45 (49)	1.96 (1.30‐2.96)	**.001**		
Hyperlipidaemia, n (%)	88 (54)	50 (54)	0.96 (0.63‐1.44)	.827		
Arterial hypertension, n (%)	155 (96)	88 (96)	1.02 (0.37‐2.77)	.974		
Heart rate, (beats/min)	70 ± 13	74 ± 16	1.01 (1.00‐1.03)	.058		
% of predicted 6MWD, %	78 ± 23	60 ± 24	0.98 (0.97‐0.98)	**<.001**	0.98 (0.97‐0.99)	<.001
Sleep apnoea, n (%)	13 (8)	13 (14)	1.51 (0.84‐2.71)	.170		
COPD, n (%)	44 (27)	38 (41)	1.71 (0.98‐2.97)	**.032**		
NYHA class ≥ III, n (%)	86 (53)	78 (85)	3.41 (1.93‐6.02)	**<.001**		
NT‐proBNP, pg/mL	780 (390 to 1440)	1940 (1150 to 2860)	1.11 (1.06‐1.16)	**<.001**		
Glycated haemoglobin, %	6.4 ± 4.4	6.2 ± 1.2	0.98 (0.90‐1.08)	.695		
eGFR, mL/min/1.73 m^2^	64 (50 to 80)	53 (40 to 65)	0.98 (0.97‐0.99)	**<.001**	0.98 (0.97‐0.99)	.030
Gamma‐glutamyl‐transferase, U/l	33 (22 to 55)	49 (29 to 104)	1.00 (1.00‐1.00)	**<.001**		
Echocardiographic parameters						
LA diameter, mm	62 ± 8	64 ± 7	1.03 (1.00‐1.05)	**.042**		
LV diameter, mm	44 ± 5	44 ± 6	0.99 (0.96‐1.03)	.708		
RA diameter, mm	61 ± 9	65 ± 8	1.03 (1.01‐1.05)	**.012**		
RV diameter, mm	36 ± 7	39 ± 8	1.06 (1.03‐1.08)	**<.001**	1.05 (1.02‐1.08)	<.001
Interventricular septum, mm	13 ± 3	13 ± 2	0.98 (0.91‐1.07)	.697		
E/E' ratio	13.8 (10.6 to 18.9)	15.0 (10.2 to 18.0)	1.03 (0.97‐1.09)	.365		
LV ejection fraction, %	59 ± 7	59 ± 7	1.00 (0.96‐1.05)	.885		
Systolic PAP, mm Hg	45 (54 to 69)	61 (51 to 75)	1.02 (1.01‐1.03)	**.002**		
Cardiac magnetic resonance imaging parameters						
LV end‐diastolic diameter, mm	47 ± 6	47 ± 6	1.00 (0.96‐1.05)	.906		
RV end‐diastolic diameter, mm	40 ± 7	42 ± 8	1.04 (1.01‐1.08)	**.009**		
Interventricular septum, mm	11 ± 2	11 ± 2	1.03 (0.91‐1.15)	.681		
LA diameter, mm	64 ± 9	69 ± 9	1.05 (1.02‐1.08)	**.001**		
LA area, cm^2^	28 (24 to 34)	31 (28 to 36)	1.03 (1.01‐1.05)	**.018**		
RA diameter, mm	64 ± 9	68 ± 10	1.04 (1.01‐1.07)	**.010**		
RA area, cm^2^	27 (22 to 34)	30 (25 to 35)	1.03 (1.00‐1.05)	**.025**		
LV ejection fraction, %	63 ± 11	62 ± 12	1.00 (0.98‐1.02)	.888		
LV end‐diastolic volume, mL	117 (103 to 142)	124 (99 to 150)	1.00 (1.00‐1.01)	.667		
Cardiac output, l/min	5.6 ± 2.9	5.3 ± 1.9	0.96 (0.85‐1.09)	.561		
RV ejection fraction, %	54 ± 11	49 ± 11	0.97 (0.95‐0.99)	**.005**	0.97 (0.95‐0.99)	.017
RV end‐diastolic volume, mL	136 (113 to 169)	155 (121 to 205)	1.00 (1.00‐1.00)	.577		
Native T1 time myocardium, ms	419 (371 to 461)	407 (354 to 471)	1.00 (1.00‐1.00)	.788		
MOLLI‐ECV	29.1 ± 3.4	31.3 ± 5.8	1.09 (1.02‐1.18)	**.016**		
Invasive haemodynamics						
Systolic PAP, mm Hg	48 (39 to 59)	55 (47 to 69)	1.02 (1.01‐1.03)	**<.001**		
Diastolic PAP, mm Hg	21 (17 to 25)	24 (19 to 30)	1.05 (1.02‐1.08)	**<.001**	1.05 (1.02‐1.08)	.001
Mean PAP, mm Hg	32 (26 to 37)	36 (31 to 43)	1.04 (1.02‐1.06)	**<.001**		
PAWP, mm Hg	19 ± 6	22 ± 6	1.06 (1.02‐1.10)	**.002**		
LV end‐diastolic pressure, mm Hg	20 ± 6	21 ± 6	1.01 (0.97‐1.06)	.517		
TPG, mm Hg	12 (9 to 17)	14 (10 to 20)	1.03 (1.01‐1.06)	**.022**		
Diastolic pressure gradient, mm Hg	1 (−1 to 5)	2 (−1 to 5)	1.02 (0.98‐1.07)	.232		
CO thermodilution, l/min	5.3 ± 1.3	5.2 ± 1.4	0.97 (0.82‐1.14)	.671		
PVR, dyn‐s‐cm^−5^	196 (143 to 252)	223 (155 to 334)	1.00 (1.00‐1.00)	**.008**		
Pooled multivariate analysis						
Persistent AF					**2.44 (1.31‐4.54)**	**.005**
% of predicted 6 MWD					**0.98 (0.97‐0.99)**	**.011**

Abbreviations: 6MWD, 6‐minute walk distance; ACE, angiotensine converting enzyme; AF, atrial fibrillation; AT II, angiotensin II; CO, cardiac output; COPD, chronic obstructive pulmonary disease; E, early mitral inflow velocity; E', early diastolic mitral annular velocity; eGFR, estimated glomerular filtration rate; HMG‐CoA, 3‐hydroxy‐3‐methyl‐glutaryl‐coenzyme A; LA, left atrial; LV, left ventricular; MOLLI‐ECV, modified Look‐Locker inversion recovery sequence‐derived extracellular volume; NT‐proBNP, N‐terminal prohormone of brain natriuretic peptide; NYHA, New York Heart Association; PAP, pulmonary arterial pressure; PAWP, pulmonary artery wedge pressure; PVR, pulmonary vascular resistance; RA, right atrial; RV, right ventricular; TPG, transpulmonary pressure gradient.

However, after pooled multivariate Cox regression analysis, only persistent AF (*P* = .005) and 6MWD (*P* = .011) remained independently associated with cardiovascular outcome.

## 4 DISCUSSION

In the present study, persistent AF was found in nearly 50% of patients. Persistent but not paroxysmal AF was independently associated with event‐free survival. Furthermore, persistent AF was closely related to important markers of disease severity such as NYHA functional class, serum NT‐proBNP, atrial dilatation, cardiac output and presence of COPD.

Several prior studies have addressed the prognostic influence of AF on HFpEF.^3^, ^6^, ^19^, ^20^ All of these studies were retrospective analyses or sub‐studies of multicentre trials. In contrast to these, we prospectively enrolled our patients, HFpEF was confirmed by invasive haemodynamic assessment, and coronary artery disease was ruled out by coronary angiography.

### 4.1 Factors associated with atrial fibrillation

We identified several parameters independently associated with persistent AF. By multivariate analysis, NYHA functional status, serum levels of NT‐proBNP, atrial dilatation, low cardiac output and COPD were associated with persistent AF (Table 2). Impaired pulmonary function and COPD have previously been linked with the development of AF.^21^ Worse NYHA status and elevated NT‐proBNP reflect advanced stages of disease, previously related to outcome in HFpEF.^22^ There are two main differences when comparing AF to SR: loss of atrial contraction, also called the booster pump function, and irregular heart rate. While the relative importance of the atrial booster pump function remains controversial,^23^ the negative influence of irregular heart rate on cardiac output has previously been described.^24^

Compared with published reference values,^25^ atrial dimensions in general were markedly increased in our study population. Furthermore, the extent of atrial enlargement was independently associated with persistent AF. In a recent sheep model of induced persistent AF, the degree of atrial dilatation and atrial fibrosis were significantly related to persistent AF.^9^ More pronounced atrial enlargement may also be linked to more advanced stages of HFpEF, indicated by significantly higher ECV values by CMR T1 mapping in the persistent AF cohort. Extracellular volume expansion plays a key role in the pathogenesis and prognosis of HFpEF.^26^, ^27^ It enhances left ventricular stiffness, which causes atrial enlargement due to increased atrial afterload. Of note, elevated ECV values did not remain significantly associated with persistent AF in the multivariate regression analysis. Patients suffering from persistent AF presented with markedly dilated right ventricles and decreased right ventricular ejection fractions, which also not remained significantly associated with persistent AF after multivariate analysis. However, impaired right ventricular ejection fraction is a known predictor for worse cardiovascular outcome^28^, ^29^, ^30^ and an association between AF and right heart dysfunction has previously been described.^29^ Interestingly, obesity, a well‐known risk factor for AF development,^4^ was not associated with AF in the present cohort.

### 4.2 Association of atrial fibrillation with outcome

Our data indicate a strong and independent association of persistent AF with cardiovascular outcome in HFpEF. Most studies that investigated AF and heart failure included both HFpEF and heart failure due to reduced ejection fraction populations and thus compared AF in HFpEF with AF in heart failure in reduced ejection fraction rather than with HFpEF in sinus rhythm.^6^, ^20^ Thus, previous data regarding the association of AF with all cause mortality or mortality and hospitalization for heart failure in HFpEF patients are limited and, moreover, report conflicting results.^3^, ^19^, ^31^

Our findings are in line with a previous large, population‐based study that showed AF to be associated with increased mortality after adjustment for age, sex, body size, kidney function, hypertension, COPD, angiotensin receptor‐, ß‐blocker and statin use.^3^ On the contrary, in I‐PRESERVE AF was associated with outcome only in the univariate analysis but was not among those demographic, clinical and biological variables that provided consistent independent prognostic information.^31^ In the present study, no differences in outcome were observed between patients in SR and patients presenting with paroxysmal AF. Thus, failure to show an association of AF with event‐free survival in the aforementioned study may also be related to the fact that paroxysmal and persistent AF patients were not analysed separately.^31^

### 4.3 Limitations

This was a single‐centre study, thus a centre‐specific bias cannot be ruled out although single‐centre studies have several advantages such as homogenous patient selection, continuous workflow and constant follow‐up. As continuous electrocardiogram monitoring by implantable loop recorders was not provided, asymptomatic AF episodes may have been missed and real‐life burden of AF episodes might be higher. In incident AF, we observed a high rate of direct onset of persistent AF. This rate may be overestimated as persistent AF might be preceded by asymptomatic and not documented short episodes of paroxysmal AF. The fact that no differences with regard to baseline characteristics and outcome were observed between patients in SR and with paroxysmal AF may be due to the low rate of paroxysmal AF in the present study. Functional imaging parameters such as ejection fraction and cardiac output on CMR may be influenced by heart rate and irregular heart rhythm. However, baseline heart rate was 73/min in persistent AF patients and did not differ significantly from patients with paroxysmal AF or SR. The cross‐sectional observational study design limits conclusions about cause—effect relationships.

## 5 CONCLUSIONS AND CLINICAL PERSPECTIVE

AF is a frequent finding in HFpEF and may affect more than 60% of patients in the course of their disease. In the present study, persistent AF was the predominant AF subtype. It was closely and independently related to event‐free survival and related to NYHA class, NT‐proBNP, atrial size, cardiac function and COPD. Up to now, no specific treatment has been approved for HFpEF patients.^2^ Similarly, effective antiarrhythmic treatment options are limited for patients with persistent AF and no survival benefit has been documented for treatments targeting rhythm control.^32^ Only the modulation of associated risk factors such as obesity has so far shown significant improvement for both HFpEF and AF patients.^33^, ^34^ Besides pharmacologic trials, future representative studies investigating dedicated antiarrhythmic treatment of HFpEF patients are highly warranted.

## CONFLICT OF INTEREST

None.

## AUTHORS' CONTRIBUTIONS

RS and JM were responsible for concept and drafting of the manuscript; FD, AAK, SA, CB, CZ‐T, MK, LF FXR, and DB performed data collection, interpretation and approved the final version.
